# What Difference Does Patient and Public Involvement Make and What Are Its Pathways to Impact? Qualitative Study of Patients and Researchers from a Cohort of Randomised Clinical Trials

**DOI:** 10.1371/journal.pone.0128817

**Published:** 2015-06-08

**Authors:** Louise Dudley, Carrol Gamble, Jennifer Preston, Deborah Buck, The EPIC Patient Advisory Group, Bec Hanley, Paula Williamson, Bridget Young

**Affiliations:** 1 Department of Biostatistics, University of Liverpool, Liverpool, United Kingdom; 2 NIHR Clinical Research Network: Children, Coordinating Centre, University of Liverpool, Department of Women's and Children's Health, Liverpool, United Kingdom; 3 TwoCan Associates, Brighton, United Kingdom; 4 Department of Psychological Sciences, University of Liverpool, Liverpool, United Kingdom; University of Edinburgh, UNITED KINGDOM

## Abstract

**Background:**

Patient and public involvement (PPI) is advocated in clinical trials yet evidence on how to optimise its impact is limited. We explored researchers' and PPI contributors' accounts of the impact of PPI within trials and factors likely to influence its impact.

**Methods:**

Semi-structured qualitative interviews with researchers and PPI contributors accessed through a cohort of randomised clinical trials. Analysis of transcripts of audio-recorded interviews was informed by the principles of the constant comparative method, elements of content analysis and informant triangulation.

**Results:**

We interviewed 21 chief investigators, 10 trial managers and 17 PPI contributors from 28 trials. The accounts of informants within the same trials were largely in agreement. Over half the informants indicted PPI had made a difference within a trial, through contributions that influenced either an aspect of a trial, or how researchers thought about a trial. According to informants, the opportunity for PPI to make a difference was influenced by two main factors: whether chief investigators had goals and plans for PPI and the quality of the relationship between the research team and the PPI contributors. Early involvement of PPI contributors and including them in responsive (e.g. advisory groups) and managerial (e.g. trial management groups) roles were more likely to achieve impact compared to late involvement and oversight roles (e.g. trial steering committees).

**Conclusion:**

Those seeking to enhance PPI in trials should develop goals for PPI at an early stage that fits the needs of the trial, plan PPI implementation in accordance with these goals, invest in developing good relationships between PPI contributors and researchers, and favour responsive and managerial roles for contributors in preference to oversight-only roles. These features could be used by research funders in judging PPI in trial grant applications and to inform policies to optimise PPI within trials.

## Introduction

Patient and public involvement (PPI) or stakeholder engagement in research refers to the practice of patients, members of the public and researchers working together to prioritise, plan, conduct and disseminate research. Recently PPI has also been introduced within the peer review process[1]. The idea behind PPI is that research is conducted *with* or *by* patients and members of the public[2], not solely *on* them. The role of patients and the public therefore extends far beyond that of a research “subject” or participant. While several different terms have been used to refer to the patients and members of the public who take on these roles, most commonly “PPI representative” or “research partner”, here we use the term “PPI contributor”. This is to avoid implying either that the small number of individuals typically involved in research can represent the diversity of perspectives among patients and the public, or that the role of PPI contributors can always be described as a partnership. Health research funding bodies strongly encourage researchers to implement PPI at every stage of the research process[3–7] and specifically to include PPI contributors on study steering committees[8–9]. This may discourage researchers from considering what PPI is really needed. PPI in research has been justified in two main ways: normatively on moral, ethical or political grounds consistent with slogans like “nothing about us without us”[10,11], and substantively in terms of the PPI potential to benefit research[10]. Normative imperatives for PPI are sometimes viewed as sufficient justification regardless of any substantive impact PPI might have on research[12]. However, PPI requires time and resource[13] and it therefore warrants scrutiny and evaluation[14].

There are indications that PPI can have favourable impacts upon every stage of the research process[13,15–19] by helping to ensure that research funds are appropriately prioritised, that research evidence is relevant to patients, and by improving recruitment and retention rates and supporting the uptake of research in practice. Indications that PPI may have unfavourable impacts upon research[15,20] or no impact at all[21] have also appeared. In this intensely moral and political arena, the rarity of such reports has raised concerns that the benefits of PPI have been selectively reported[13,22], and led to questions about the quality of the evidence-base for PPI in trials. Problems with the conceptualisation and measurement of the impact of PPI have also been identified[23] and few studies have accessed the perspectives of both PPI contributors and researchers. Moreover, much of the literature on the impact of PPI in research has not focussed specifically on randomised clinical trials (RCTs), although these are regarded as particularly likely to benefit from PPI[13].

In the context of EPIC (Evidence base for Patient and public Involvement in Clinical trials), a mixed methods investigation of a cohort of trials, we have previously reported on PPI in the early stages of trial design[24], the implementation of PPI[25], and on training in PPI for researchers and PPI contributors[26]. In this paper we report on findings from our qualitative study about what impact PPI had within the trials, and the factors that influenced its impact. To address some of the limitations of the existing evidence we examined both PPI contributors’ and researchers’ accounts, some of whom had worked on the same trials, so that we could corroborate their accounts. We also sampled from trials involving a range of conditions with the aim of accessing informants with a diversity of views and experiences of PPI.

## Methods

### Design

As noted above, our qualitative study was one of a number of work streams within the EPIC project, which investigated PPI in a cohort of RCTs funded by the United Kingdom National Institute for Health Research (NIHR) Health Technology Assessment (HTA) programme between 2006 and 2010. These work streams included a quantitative study of data extracted from documents (mainly grant application forms and trial protocols) for each of these RCTs describing what PPI was planned, and surveys of chief investigators (CIs) or another senior member of the research team, PPI contributors and trial managers (TMs) on their views and experiences of PPI within these trials. For the qualitative study we invited CIs, PPI contributors and TMs who had responded to the survey to be interviewed. By allowing us to access informants’ accounts of PPI in their own words and to analyse them inductively, the qualitative study aimed to complement the other EPIC work streams. Given the moral and political expectations surrounding PPI, we thought it was particularly important to adopt an interpretive approach[27,28] and consider how informants talked about PPI as well as the content of their accounts. Therefore we considered the language informants used to describe PPI and the aspects of PPI they gave little emphasis to in their interviews as well as what they emphasised. Before each interview we reviewed the plans for PPI in the documents for each trial in order to tailor our questions and identify particular lines of enquiry to pursue. The interviews enabled us to seek clarification and prompt informants to elaborate on their experiences and perspectives. Similarly, informants were able to seek clarification from us, to elaborate on their perspectives and to raise topics that we had not anticipated. EPIC had its own patient advisory group; we advertised for, interviewed and appointed the members based on their experience as patients and carers, or as PPI contributors within RCTs.

### Ethics statement

Our original ethics application specified that written consent would be taken. At the time of applying for approval (University of Liverpool Research Ethics Committee Ref: RETH000489), we expected that the audio-recorded interviews would be face to face and written consent would be obtained prior to the start of the interview. However, when we contacted individuals who had expressed an interest in participating in the study they all requested to be interviewed over the phone. In line with the ethics approval, prior to the interviews we had sent participant information sheets and forms out to participants to obtain their written consent but a number of participants did not return the consent forms by the arranged interview date. When the consent forms had not been received prior to the interview date/time we audio-recorded their consent. This was a change to the particular method of obtaining informed consent as described in the ethics application, but consent was still obtained from all participants and audio-recorded. When this change to the consent method came to light we informed the ethics committee. They stated that although they could not give approval for the change retrospectively they were assured that all participants had provided consent and that this was documented via the audio-recordings.

### Recruitment and sampling

We contacted CIs using the email address provided on the trial grant application form. We initially sampled CIs for maximum diversity based on their survey responses (S1 File), although we eventually invited for interview all but three of the CIs who had responded to the survey and indicated an interest in the qualitative study. We aimed to access PPI contributors by: 1) asking CIs to send PPI contributors an invitation from the EPIC research team; 2) sending invitations to PPI contributors via the chairpersons of the steering committees for the trials in our cohort; 3) placing advertisements on PPI related websites[29,30] listing the particular trials. We obtained contact details for TMs from clinical trial units, trial websites and protocols, or via CIs. We invited all PPI contributors for interview who returned a survey response and indicated willingness to take part in the qualitative study. We invited TMs for all trials for which the CI or PPI contributor had been interviewed. We sent potential informants an email and information leaflet, with the reasons for doing the research; to generate a detailed understanding of PPI in RCTs and to explore the experiences of those involved, and inviting them to contact the EPIC research associate if they wished to be interviewed. Non-responders were sent two reminder emails. We anticipated that some PPI contributors may access their email accounts infrequently so we subsequently telephoned those who had not responded.

### Interviews

LD, a psychologist with experience and training in conducting and analysing qualitative interviews, conducted audio-recorded semi-structured telephone interviews with informants between April 2013 and November 2013. Before starting interviews she explained that study data would be anonymised and kept confidential. Interviewing was conversational to allow informants to freely voice their views and experiences. LD adopted a neutral stance in her interviewing to avoid creating a sense that informants’ had to justify or defend their approach to PPI, which may have inhibited or coloured their accounts. She also familiarised herself with each of the documents for each trial before interviews, to tailor questions within the interview topic guides to particular trials. We developed three versions of the topic guides as appropriate for each of the three informant groups (CI, PPI contributor and TMs), although the versions mirrored one another to ensure core topics were explored. Topic guides were reviewed by the patient advisory group and developed in the light of the on-going data analysis (S2 File summarises the topic guides). Interviewing paralleled the analysis and continued until theoretical saturation[31] had been reached and additional data ceased contributing to the analysis. Interviews were transcribed using an “efficient” verbatim style that involved transcribing the content of informants’ accounts, rather than detailed features of speech such as sub-vocalisations and duration of pauses and hesitations. LD anonymised all transcripts and checked them for accuracy.

### Analysis

Analysis was informed by the principles of the constant comparative method[32,33] with elements of content analysis[34]. We used procedures to support rigour in qualitative research[35]. To ensure a contextualised analysis we referred to transcripts as a whole as well as to particular data segments. We initially analysed CI and PPI contributor transcripts at the informant group level for evidence of their beliefs and experiences about the process and impact of PPI. Subsequently, we triangulated CI and PPI contributor transcripts within each trial, before comparing them with the TM transcripts within each trial. Where trials did not have a full data set (i.e. did not include all three groups of informants), we compared the two available accounts. Where only one account was available, analysis was at the informant group level.

LD led the analysis, reading CI and PPI contributor transcripts several times before developing open codes. BY also read multiple transcripts and she and LD met regularly to compare interpretations of the data and review the ongoing analysis. Open coding took place at multiple levels from line-by-line coding of detailed descriptions to the general stance informants took towards PPI. Open codes were grouped into categories and organised into a framework. Coding and indexing of data was assisted by NVivo 9 software, and we continually compared categories to new data and amended them to ensure they reflected the data while accounting for deviant cases. For TM transcripts we open coded sections relevant to the categories emerging from the CI and PPI contributor analysis. Subsequent discussion and review of detailed analysis reports by other members of EPIC team (comprising individuals with backgrounds in biostatistics, psychology, sociology, academic research on PPI, and experience as patients, carers, PPI contributors and researchers who have implemented PPI in health research and clinical trials), helped to refine the analysis and corroborate the findings. This included DB who led an analysis of the same dataset on the implementation of PPI[25]. To evidence our interpretations we present illustrative extracts from the data. Extract codes indicate informant group (CI, PPI, TM) and trial identification numbers. Where more than one PPI contributor was interviewed for the same trial, we indicate 1 or 2.

## Results

### Sample

We recruited informants from 28 trials (S3 File). For nine trials we interviewed both the CI and PPI contributor, and for five of these trials we interviewed the TM too. Appendix B (in S2 File) also indicates the setting of the trials and the type of intervention under investigation.

**Fig 1:**
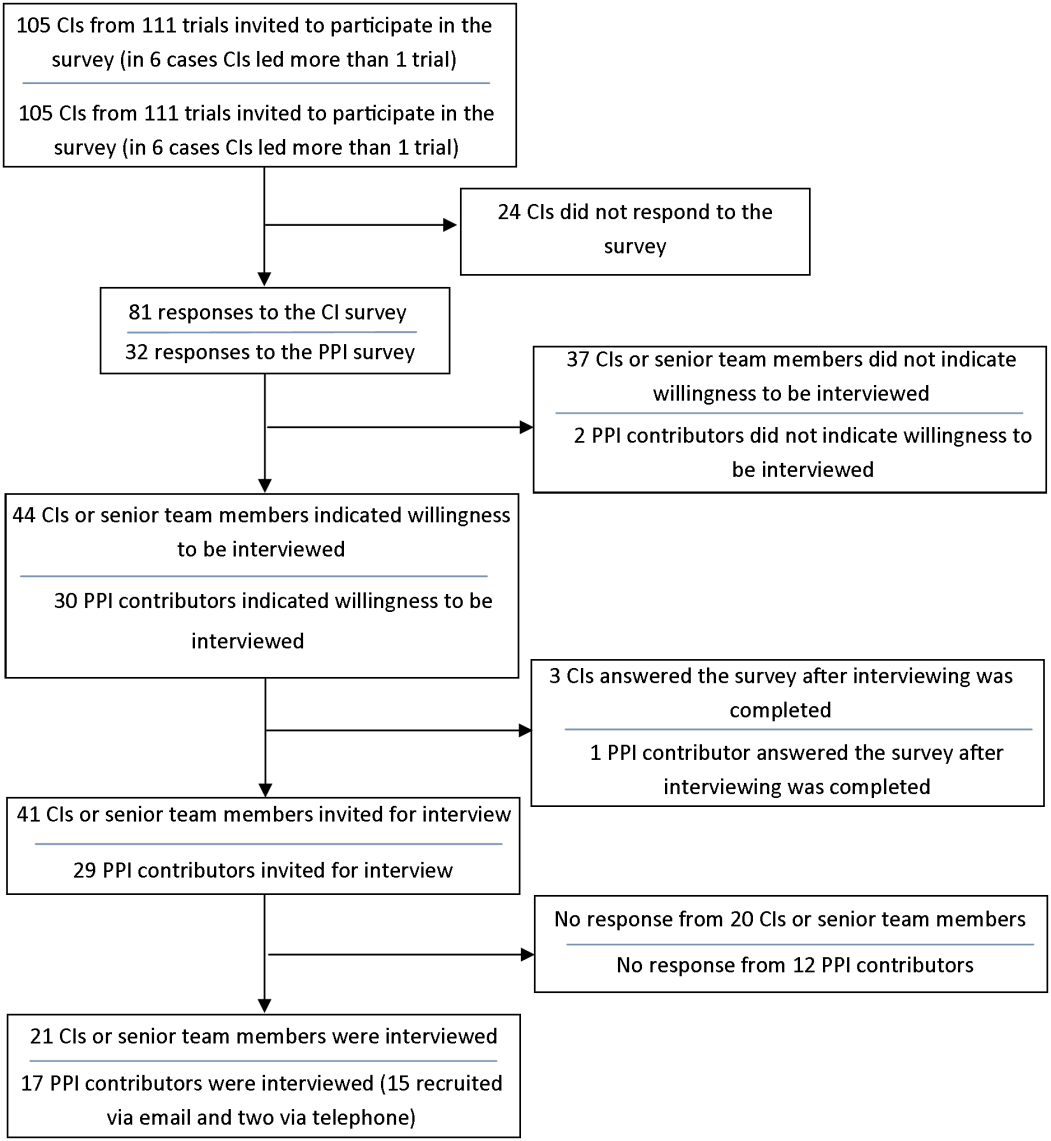
Flow diagram illustrating CI and PPI contributor recruitment.

The flow diagram in Fig 1 illustrates the recruitment of CIs and PPI contributors. Of those invited for interview, 21/41 (51%) of CIs and 17/29 (59%) of PPI contributors participated. Regarding TMs, out of the 28 participating trials, one trial did not have a TM at the time of our study and we were unable to obtain contact details for TMs within three trials. We invited the remaining 24 TMs for interview; of these, nine did not respond, five declined and 10 (42%) were interviewed.

Appendix A (in S1 File) shows the survey questions that informed initial sampling of CIs and compares responses to these questions for the qualitative interview sub-sample compared to the wider sample of surveyed CIs. Of the 32 PPI contributors who responded to the survey, 31 were accessed via CIs and one via the chairperson of a trial steering committee. We did not recruit any PPI contributors through the online advert. Interviews lasted 45 minutes on average.

### Impact

None of our informants identified PPI as having an unfavourable impact on the trial. Of the 21 CIs interviewed, 14 described PPI as having an impact and seven explicitly stated that they felt PPI had not made an impact. Of the 17 PPI contributors, 11 reported that they felt their input had made a difference to the trial; three explicitly indicated that they had made no impact and three could not identify impacts arising from their input. Of the 10 TMs, five described PPI as having made an impact and five did not identify any impacts of PPI. When we triangulated the accounts of researchers and PPI contributors from the same trial, they were largely in agreement about the perceived impact of PPI. Divergences between the groups could largely be attributed to informants being unable to remember specific PPI contributions because a trial had started “a long time ago”.

Based on the accounts of informants who perceived PPI as having an impact, we distinguished two main types; focussed and diffuse (S4 File). Focussed impact comprised PPI contributors’ input that, from the perspective of the informant, changed or influenced an aspect of the trial, whereas diffuse impact comprised PPI contributions that influenced the way researchers thought or felt about the trial. In addition to the examples in Appendix D (in S4 File), focussed impact included PPI contributors helping to choose the primary outcome for the trial and to increase recruitment through their contacts and networks. As Appendix D (in S4 File) indicates, diffuse impact largely entailed interactions between researchers and PPI contributors that helped to reassure the research team and increase or maintain their confidence and motivation for the trial. For example, both researchers and PPI contributors described how a PPI contributor’s presence kept the research “grounded” and reminded them “what it’s about.”

Informants who reported no impact either explicitly stated that they felt PPI had not had an impact, or they were unable to identify any impacts arising from PPI.

“Within that TSC I can’t remember them making any particular contribution that changed the way we run the, ran the study. And if they had made a substantial contribution I would remember.”

(CI—10)

“She reviewed all the paperwork, she came to the meetings, but did she actually change the trial in a meaningful way? Well no um probably not.”

(CI—7)

Informants who explicitly stated that PPI had not made an impact spoke only of the lack of focussed impact. None of these informants referred to diffuse impact when speaking about the absence of PPI influence. In contrast, many informants who reported that PPI had made an impact identified both the focussed and diffuse types. As their accounts did not deem one type more important than the other, we use the term “impact” to refer to both types.

We identified two main influences on whether informants perceived PPI as having an impact; whether CIs expressed any personal goals and plans for PPI aside from its perceived role in leveraging funding, and the quality of the relationship between the PPI contributors and the researchers.

### Goals and plans

CIs who reported having goals and plans tended to perceive PPI as having an impact, and PPI contributors on the same trials corroborated this by describing the differences that their involvement had made. CIs’ goals and plans for PPI included their ideas about what they wanted to achieve by including PPI in the trial (beyond complying with funders’ requirements), what the contributors’ role would be and what activities PPI contributors would be involved in. For example, one CI expected PPI to:

“Input into the choice of measures used to evaluate the intervention […] the tools to make sure that they’re workable, that they capture the things that are important to the service users, and I’d expect it to help with interpreting the findings.”

(CI—3)

Eleven CIs reported having goals and plans for PPI and seven of these felt PPI had had an impact. Nine reported having no goals and plans and only two of these felt PPI had an impact. CIs’ goals and plans also linked to their ideas about how to choose PPI contributors (for example, should the PPI contributor be a patient or charity member and how experienced they should be) and what stage(s) of the trial the PPI contributors should be involved in. Researchers who reported impact, explained how PPI needed “to be done in a way that means it’s central” (TM—3) to the trial, and emphasised the importance of planning what they wanted to achieve from PPI and how to achieve it.

“Planning it from the beginning really. So thinking what do we really want to get out of this, what input do we want to have and how do we want to involve them, um, because if you can involve them in more of the trial […] get more input from them and make it more meaningful, then you’re going to get sort of a better outcome.”

(TM—27)

In contrast, researchers who lacked goals or plans for PPI spoke of their lack of experience of PPI and limited understanding of the function and process of PPI. Their motivation to include PPI was primarily to comply with the requirements of funding and they gave little priority to considering what PPI might achieve or how to implement it. Some also openly reflected on how, by not having any goals or plans for PPI in their trial, they could be accused of “tokenism” or of following a “tick-box” approach.

“It was a degree of tokenism and I say that completely openly that we felt we should […] actually none of us knew quite why or what the patient’s role would be or how it would work out […] there was a patient who we knew who’d already had the injury that we were seeing, so let’s get them involved.”

(CI—5)

“If I’m honest, I don’t think there was much planning for PPI. I think it was really more a matter of when we were putting together the trial steering committee, we said, “oh we should have a patient representative, let’s see who we can find”.”

(CI—6)

These two CIs did not report any impact from PPI. While commenting that PPI had made little difference within their trials, researchers also acknowledged, vaguely and in some cases reluctantly, that their interactions with PPI contributors had been a “positive experience” or they implied that the research team could have done more to try to ensure that their trial was able to benefit from PPI.

“It’s required by the funder and therefore it would help me achieve my goal of getting the study funded. Um, it’s clearly politically required […] we’ve had fairly positive um, a very positive experience of the patient reps […] but I don’t think [they] have added very much. But they’re there, we can say they’re there […] ticking a political box.”

(CI—2)

“To be honest, we probably didn’t think about it and discuss it as much as maybe we should have to get the full, utilise it as much as we possibly could. I think our funders, we have to have a lay member as part of our trial steering committee.”

(TM—5)

How CIs spoke of the value of PPI seemed to be related to whether they had goals for it. For example, a CI whose goal was for PPI to support recruitment, emphasised how important it was to researchers working in a particular disease area to engage with patients with that disease: “one of the things that I’ve tried to convey is that I think that when you are studying pathology, to talk to the patients that have that pathology is extremely important” (CI—21). This account stood in marked contrast to others who described PPI as “political correctness” and reported having no goals beyond using it for “getting the study funded”.

“It’s almost become an industry, um and I think everybody has just joined on in saying you need PPI for all of these studies. I have to say I’m not convinced.”

(CI—2)

Researchers whose accounts indicated they did not value or have goals for PPI tended to implement it mainly in one way—by including PPI contributors on trial steering committees. We elaborate in the next two sub-sections on how the roles of PPI contributors and the stage of the trial at which PPI was implemented seemed to mediate the relationship between PPI goals and plans and the perceived impact of PPI.

#### Oversight, managerial and responsive PPI

Researchers identified several different roles for PPI contributors. We grouped these into three main types: oversight, managerial and responsive. An oversight role usually entailed one PPI contributor having formal involvement in the trial on multiple but infrequent occasions, for example as members of trial steering committees or data monitoring committees meeting six monthly or annually. A managerial role was also usually formal and entailed one PPI contributor being involved on a more regular basis, for example as a co-investigator or as a member of the trial management group. Responsive PPI was often impromptu and more informal than the other two types and often involved researchers approaching PPI contributors as difficulties arose: “when we had a problem, I went back to her and said, can you please comment on these questionnaires because it’s taking too long for people to fill them in” (CI—3). Responsive PPI also included contributors advising on patient information sheets, troubleshooting recruitment difficulties and tailoring interventions, methods of data collection and follow-up to the needs of patients. Of the 21 trials for which a CI was interviewed, eight implemented one type of PPI, nine implemented two types and four implemented three types giving 38 instances of PPI across these trials. Each CI described the perceived impact for each type of PPI they implemented.

In general, researchers who had experience of more than one type of PPI tended to favour informal, responsive PPI. For example, a CI spoke of how “formal” PPI, where PPI contributors had an oversight role, did not work as well as “informal” PPI, where researchers approached PPI contributors on a responsive or “as required” basis.

“Comparing the two trials that we have, where we have formal and informal, I do think so, the informal arrangements worked very well.”

(CI—1)

Out of the 21 trials, nine included responsive PPI and researchers from six of these trials felt that this type of PPI had an impact. While researchers tended to associate responsive PPI with perceived impact, this may be because they tended to have very defined goals for this type of PPI, making it easier to link PPI input and impact. Also, responsive PPI usually involved more PPI contributors than the other types and researchers emphasised the importance of accessing a “plurality of opinion” and doing so as and when questions or problems arose. Therefore, it could be that researchers associated responsive PPI with impact because it was particularly helpful in identifying strategies to address problems. It could also be that contributions from this type of PPI carried more weight with the research team because it allowed access to a more diverse range of contributors who researchers saw as more “representative” of the target population, compared to the PPI contributors who had oversight and managerial roles.

“Rather than just having members of the committee […] having more lay reference groups that we can refer to […] you need a broader pool of people to advise on this to make sure you get a really sensible reality check.”

(CI—10)

PPI contributors who had oversight and managerial roles sat on committees and attended formal and scheduled meetings. While some contributors were able to recall specific contributions that they made during these meetings, researchers often struggled to pinpoint what particular individuals had contributed in meetings: “when you’ve got, um, meetings like that […] unless you did a full analysis, it would be difficult to tell what the impact was of any individual” (CI—3). Nevertheless, managerial PPI was associated with impact more often than oversight PPI. Out of the 21 trials for which a CI was interviewed, 11 included managerial PPI and researchers from seven of these trials reported that they felt PPI this type of PPI had made a difference. In contrast, of the 18 trials that included oversight PPI, researchers from five trials reported that they felt this type of PPI had made a difference. Aside from the lack of goals that often accompanied oversight PPI, its relative lack of impact could be linked to the infrequent contact between the PPI contributors and the research team, whereas managerial PPI contributors would usually be present when particular problems were discussed and could offer immediate advice.

#### Stage of PPI implementation

Both researchers and PPI contributors pointed to how it was important to implement PPI early during the course of a trial before plans were “set in stone” and so that PPI contributors had opportunities to develop a sense of “ownership” of the trial. We categorised early involvement as PPI activity that was implemented around the time that the first meetings about a trial were held and before the final funding application was submitted. Of the 28 trials, 16 had PPI contributors that had been involved at an early stage, four had PPI involvement after funding only and eight had a mixture of some PPI contributors being involved at an early stage and others joining after funding was confirmed. Informants were less likely to report impact from PPI that had been implemented after funding compared to PPI implemented prior to funding. Both researchers and PPI contributors emphasised how contributors were better placed to contribute at an early stage and also towards the end of a trial, whereas opportunities to influence a trial mid-course were seen as limited.

“It's more just that my involvement was probably more useful at the beginning, and I think it will probably be more useful at the end when we get into the interpretation of data and how it's going to be probably disseminated. But the middle, the middle bit is fairly technical.”

(PPI—9)

“Decisions may have been made that are difficult to change [after funding] that, from a patient’s perspective, may be wrong.”

(PPI—6)

“So more input at the beginning in the choosing the research question and at the end in terms of dissemination, and less actually in the day to day running of the management of the trial, which for most patients is um, I think it was a bit of tokenism.”

(CI—5)

### Relationships

Both CIs and PPI contributors spoke of how it was important to invest time and effort in forming a relationship so that contributors felt part of the team. In turn, PPI contributors who reported feeling part of a team tended to report an impact from their involvement, in contrast to those who did not feel part of the team. Of the 17 PPI contributors, seven indicated or implied that they felt part of the team, all of whom felt their involvement made a difference. Of 10 PPI contributors who explicitly stated or implied they did not feel part of the team, only four felt their involvement had made a difference. PPI contributors who felt they were part of the team tended to describe their relationship with researchers as a “partnership” and referred to being “treated as equals” (PPI—7). Both CIs and PPI contributors spoke of how feeling part of a team empowered PPI contributors to voice their perspectives in interactions with the research team.

“Build a relationship with them a little bit as well so that they are comfortable and confident. Because I think probably it could be a little bit daunting, sitting around a table with a whole pile of professionals and experts.”

(CI—6)

“It was actually quite an engaged process and I felt very much part of the team, rather than just somebody sitting on the outside who occasionally was asked for their view. So I felt I was very able to sort of shape and steer the project as well.”

(PPI—3)

The stage of the trial that PPI contributors became involved, and the frequency of their contact with the research team, influenced whether or not PPI contributors felt a part of the team. As noted above, PPI contributors described how they could make more of a contribution when they were involved in the early stages of a trial, whereas becoming involved later in the course of a trial made it hard for PPI contributors to develop a relationship with the research team.

“If a patient comes in at a later stage, the group has jelled so […] you could become an outsider because the group is already formed.”

(PPI—6)

One PPI contributor, who did not identify any impacts arising from his role within the trial he was currently involved in, compared that particular trial, where his involvement had not started until after the funding application had been submitted, to other trials where he had been involved from the beginning.

“If you come in late, or pick up from someone else, then it's maybe not as easy as whether it's something that you've been involved in from the application stage forward, and the relationships are formed.”

(PPI—9)

Similarly, a CI who could not identify any impact of PPI and was “never convinced” that the PPI contributors “felt” part of the team commented:

“I think it's much harder to actually express their views as they go along and it doesn't feel like a genuine partnership.”

(CI—12)

## Discussion

### Summary of findings

Our study is the first to provide insights from a diverse sample of researchers and PPI contributors about the pathways to impact for PPI within RCTs. Well over half of the informants indicated that PPI had made a difference to the trial or influenced the trial team and none reported unfavourable impacts from PPI. CIs who described goals for PPI and planned its implementation in the light of these goals tended to report impact, whereas those whose goals for PPI did not extend beyond meeting perceived funding requirements usually reported little or no impact from PPI. PPI contributors who spoke of having a good relationship, particularly in terms of feeling part of the team, also tended to report impact from PPI, and both researchers and PPI contributors pointed to the importance of implementing PPI before seeking funding. Despite the frequent practice and policy recommendation[8,9] to include PPI contributors on steering committees, researchers and PPI contributors often reported that such oversight roles made little or no difference within a trial. Whether CIs valued PPI seemed to be linked to the goals they described and how they implemented PPI. CIs who expressed scepticism about PPI focussed mainly on using PPI to meet funding requirements, whereas those who valued PPI often described in detail how it was of benefit within their trials. CIs who were sceptical of the value of PPI tended to implement it only by including PPI contributors on trial steering committees. Our study confirms that some researchers seem to accord little value to PPI. It also raises the possibility that this may become a self-perpetuating cycle, with such researchers implementing PPI in ways that may provide little opportunity for it to benefit RCTs and then concluding that PPI made little difference to their trials.

### Previous work and implications of findings

To our knowledge this is the largest qualitative study of PPI in trials to date. Several of our observations receive support from previous studies and reviews of PPI, although most of this work has not concentrated on PPI in trials. Compared to other forms of health and social care research, trials are highly structured and intensely regulated entities, so the relevance of PPI studies conducted outside the context of a trial is limited for understanding how PPI can make a difference within trials. For example, it will usually be harder to change aspects of a trial after it has started compared to other types of studies. Nevertheless, previous work has pointed to the different types of impact, both focussed[12, 15–19] and diffuse[13] that we identified. Previous work has also identified the role of researchers’ values[36], the quality of the relationship between researchers and PPI contributors[14] and the importance of implementing PPI through the “life course” of a project[13] in facilitating the impact of PPI. Our findings concur with the recently published Public Involvement Impact Assessment Framework[37], which was informed by a large mixed method study of the views and experiences of members of the UK health and social care research community. This emphasised the importance of careful planning in implementing PPI and encouraged researchers to be explicit in thinking about how their approach to PPI will lead to the impacts they seek[37,38]. International guidance has also emphasised the importance of having PPI from an early stage, and having wider involvement than PPI on a steering committee[7], although such guidance has lacked an evidence base until now. Many countries now encourage or require PPI to be included in research[3–7], therefore our findings can be applicable internationally. Opportunity to compare our findings to previous evidence beyond this is limited because, as we note above, few studies have specifically investigated the impact of PPI on trials[13] and we are not aware of any studies that have examined influences on reported impact of PPI across multiple RCTs. As the first evidence to indicate the ineffectiveness of limiting the involvement of PPI contributors to oversight roles on steering committees, our findings indicate that some recommendations on PPI in steering committees need to be amended to acknowledge the limitations of this type of PPI as the sole means of engagement and will be of interest to research funders as well as PPI contributors and researchers. The trials we studied often combined two or more approaches to PPI, and our informants described the importance of having the freedom to tailor PPI to the emergent needs of their trial.

The findings are timely given the increased emphasis on stakeholder involvement in research internationally and the recent publication of the NIHR’s ‘Going the Extra Mile’ review of PPI in the UK[39]. The review points to the need for debates about the need for public involvement to mature into conversations that focus on what works. Our findings address this need, providing evidence to inform strategies to optimise PPI within trials, and thereby to maximise the potential of PPI to improve the quality of research. This review also identifies the need for training in PPI, which may be required in order to undertake the roles of PPI we have identified in our findings. The findings also point to the difficulties that funding panels and reviewers face in assessing the quality of a trial team’s plans for PPI, beyond identifying potential “red flags” such as PPI contributors being limited to steering group membership or their involvement being sought only after funding has been awarded. Pre-funding grants for PPI are available, although the funding available is often small[24] and inaccessible to those working within the tight timescales of a typical funding call. Increasing the availability and scale of resource to provide an infrastructure to support researchers and contributors to initiate PPI at the pre-funding stage would help to facilitate earlier implementation. Many researchers believed that funding would not be forthcoming unless they included PPI. While this might be regarded as indicating the success of policies to promote PPI, it was clear that some circumvented these polices by adopting a minimalistic approach to PPI. In the light of our findings research funders might want to consider how their policies could be refined to address this difficulty. Our study points to the inadvisability of applying “one size fits all” methods to the implementation and evaluation of PPI in research, and underscores the importance of funding panels conducting nuanced assessments of a research team’s goals for PPI in the context of a particular trial. This might encompass scrutiny of a research team’s account of how their proposed trial stands to benefit from PPI and assessing the suitability of their plans in the light of these goals. It might also involve accepting that PPI should be proportionate to the needs of a particular trial and that a minimal approach to PPI may be legitimate in some cases. Researchers who can adequately justify such an approach should not fear that their chances of funding success will automatically be jeopardised by being candid about this. A sizable minority of informants did not report any impact from PPI. While our findings point to problems in the implementation of PPI as contributing to this lack of perceived impact, it is conceivable that some trials will have little to gain from extensive and elaborate forms of PPI.

### Strengths and limitations

Our study had some limitations and our findings should be regarded carefully, particularly as PPI is a field where policy has tended to outpace evidence. Like most other studies exploring the impact of PPI in research, it was limited to investigating researchers’ and PPI contributors’ reports of their views and experiences[13]. Objective techniques for evaluating impact and its influences remain elusive in a process that is inherently relational, subjective and socially constructed[14]. For example, some informants reported that PPI contributors’ input helped to improve response rates by reducing the length of questionnaires, yet there is the possibility that valuable information was lost in the process. Participants in Barber’s[15] recent mixed methods Delphi survey and qualitative interview study questioned the feasibility of objectively evaluating the impact of PPI on most research processes and outcomes. In this regard, a strength of our study is that we triangulated the accounts of multiple informants in half the sampled trials. Linked to our study’s retrospective design however, informants struggled to recall particular examples of PPI input, and as others have noted[14], there are inherent difficulties in attributing impact to the contributions of particular individuals, when the actions to address many difficulties within trials are likely to be the product of a series of complex interactions among research team members. Our sample was limited to trials within one UK-based research funding stream, the NIHR HTA programme. While this limits the transferability of our findings, as one of the world’s leading funders of health research, NIHR research activity is substantial. Although our informants were drawn from a cohort of trials, we could only interview those who opted to do so. The response rate to the CI survey, which was our main route for accessing interview informants, was high (73%) while the response rate for the CI interviews was lower (51%). Also, all of the PPI contributors interviewed were involved in managerial or oversight roles as we were unable to access those in responsive roles, because most researchers did not hold contact details for contributors in such roles. Our access to the information about PPI was limited in some cases; for example in cases where the only informant from a trial was the PPI contributor in an oversight role, we were unable to ascertain the other types of PPI within that trial.

Because our interview sample was drawn from a cohort study and survey we are able to provide more information about our sample than is typical for qualitative studies. The survey responses of the interviewed CIs were slightly more favourable towards PPI than the wider group of surveyed CIs. However, the survey responses cannot be taken as a fixed or true point from which assess the adequacy of our sample[40]. In their interviews participants described their experiences in detail and we were able to consider how they talked about PPI, as well as what they said about it. In this context, it is notable that, while all of the CIs in the survey reported some impact from PPI[41], the interview accounts of CIs told a rather different story with one third describing PPI as having no impact. This indicates the usefulness of qualitative approaches for investigating complex processes like PPI, which are subject to moral, reputational and other sanctions. The diversity of perspectives that we accessed also suggests that we had some success in minimising the selectivity that has been a difficulty in some previous work on PPI. Finally, although we acknowledge the limitations of quantification in qualitative research[42,43] and emphasise that the figures we present be interpreted carefully, we have used some quantification in reporting our findings as this can help make explicit the basis for conclusions in qualitative studies[44].

### Future research and conclusions

Given the difficulties for some informants in recalling PPI contributions, future research in this area that takes a prospective approach would be valuable, although the time that many trials take from conception to completion could mean such research is prohibitively expensive. PPI is an area of rapidly evolving practice and prospective research would also be valuable to explore how such changes are influencing how PPI is interpreted and practised, particularly as the trials in our cohort were funded between eight and four years ago. In view of the difficulties for informants in attributing impact and the relational and subjective nature of PPI activity, ethnographic research that combines observation and multi-informant interviews is likely to be informative. Many will also regard future prospective investigation of the impact of PPI on trial outcomes such as recruitment, retention and participant experience of trials as essential to further optimise PPI. We conclude that if researchers, PPI contributors and research funders wish to enhance PPI in trials they should consider how PPI can inform or benefit a trial and plan PPI to suit these goals, work to develop good relationships between PPI contributors and researchers, involve PPI contributors at an early stage and favour responsive and managerial roles for PPI contributors in preference to roles that only involve oversight.

## Supporting Information

S1 File Survey responses used in initial sampling CIs for interview and comparison of responses for interview sub-sample and survey sample. (DOCX)

S2 File Summary of interview topics covered. (DOCX)

S3 File Informant interviewed, trial setting and intervention type. (DOCX)

S4 File Examples of focussed and diffuse impact. (DOCX)
