# Supplementary material for: What Difference Does Patient and Public Involvement Make and What Are Its Pathways to Impact? Qualitative Study of Patients and Researchers from a Cohort of Randomised Clinical Trials
Source: PLoS One. 2015 Jun 8;10(6):e0128817. doi: 10.1371/journal.pone.0128817 (PMC4459695; doi:10.1371/journal.pone.0128817)
Supplement: S1 File — (DOCX) [file pone.0128817.s001.docx]

**S1 APPENDIX:**

| **Table 1:- Survey responses used in initial sampling CIs for interview and comparison of responses for interview sub-sample and survey sample** | | |
| --- | --- | --- |
| **Question 1: In general what is your personal view on PPI?** | **CIs who were interviewed n(%)** | **Response distribution within CI survey n(%)** |
| PPI should always be included in a research study | 12 (57) | 42 (52) |
| PPI can be beneficial but is not always necessary | 8 (38) | 35 (43) |
| I am not convinced of the benefits of PPI | 1 (5) | 4 (5) |
| **Question 2*: What motivated you to include PPI in your trial?** |  |  |
| I think including PPI is the right thing to do | 15 (76) | 55 (70) |
| I have previous experience of the benefits of PPI | 12 (57) | 45 (57) |
| PPI was a requirement for research funding | 8 (38) | 39 (50) |
| A PPI contributor offered their help | 1 (5) | 4 (5) |
| Other | 2 (10) | 2 (3) |
| **Question 3*: Which PPI contributor/s did you involve?** |  |  |
| Patient | 14 (67) | 54 (68) |
| Carer | 2 (10) | 15 (19) |
| Parent | 2 (10) | 13 (16) |
| Charity member | 10 (48) | 24 (30) |
| Medical staff | 4 (19) | 11 (14) |
| Other | 3 (14) | 9 (11) |
| *CIs could provide more than one response to questions 2 and 3 | | |

**APPENDIX 2:**

| **Table 2: - Summary of interview topics covered** | | |
| --- | --- | --- |
|  | **Researchers** | **PPI Contributors** |
| **Expectations** | Understanding of PPI | Previous experience of being a PPI contributor |
|  | Experience of including PPI in research | Expectations about what working on the current trial would be like |
|  | Goals or plans for PPI in current trial |  |
| **What happened?** | Stage of PPI implementation | How did they become involved in the trial? |
|  | Identifying and selecting PPI contributors | PPI contributor’s role |
|  | Roles of the PPI contributors | Relationship with research team |
|  | Overall experience of including PPI in the current trial |  |
| **Impact** | Perceived contributions of PPI | Differences made to the trial as a result of their input |
|  | Challenges of including PPI | Benefits to themselves of being involved |
|  |  | Challenges of being involved |
| **Training and support** | Training or support given to PPI contributors | Training or support for their role |
|  | PPI training received by researchers | Views on PPI training for researchers |

**APPENDIX 3:**

| **Table 3:- Informant interviewed, trial setting and intervention type** | | | | | |
| --- | --- | --- | --- | --- | --- |
| **Trial** | **CI or senior team member interviewed?** | **PPI interviewed?** | **TM interviewed?** | **Setting*** | **Intervention** |
| **1** | y | Y | N | Community | Education and exercise |
| **2** | Y | Y | N | Tertiary | Device |
| **3** | Y | Y | Y | Secondary | Education |
| **4** | Y | N | N | Tertiary | Drug |
| **5** | Y | N | Y | Secondary | Surgical |
| **6** | Y | Y | N | Secondary | Exercise |
| **7** | Y | Y | N | Primary | Community care |
| **8** | Y | Y (2 PPI contributors) | Y | Tertiary | Drug |
| **9** | Y | Y | Y | Secondary | Device |
| **10** | Y | N | N | Social care | Exercise |
| **11** | Y | Y (2 PPI contributors) | Y | Secondary | Surgical |
| **12** | Y | N | N | Secondary | Device |
| **13** | Y | N | Y | Secondary | Drug |
| **14** | Y | N | N | Secondary | Surgical |
| **15** | Y | Y | Y | Primary | Exercise |
| **16** | Y | N | N | Primary | Exercise |
| **17** | Y | N | N | Secondary | Surgical |
| **18** | Y | N | Y | Primary and secondary | Exercise and community care |
| **19** | Y | N | N | Primary | Other |
| **20** | Y | N | N | Emergency | Community care |
| **21** | Y | N | Y | Secondary | Device |
| **22** | N | Y | N | Secondary | Surgical |
| **23** | N | Y | N | Secondary | Device |
| **24** | N | Y | N | Tertiary | Drug |
| **25** | N | Y | N | Emergency | Surgical |
| **26** | N | Y | N | Secondary, Tertiary | Surgical |
| **27** | N | Y | Y | Emergency | Drug |
| **28** | N | Y | N | Secondary | Device |
| ***** A primary setting is the first point of consultation for a patient within the healthcare system, for example a general practitioner. A secondary setting is care provided by a medical specialist that cannot be directly accessed by a patient, for example as a hospital outpatient. A tertiary setting is specialist consultative healthcare, on referral from primary or secondary care, that has personnel and facilities for advanced investigation and treatment, for example a specialist cardiac unit. | | | | | |

**APPENDIX 4:**

| **Table S4:- Examples of focussed and diffuse impact** | |
| --- | --- |
| **Focussed impact** | **Diffuse impact** |
| “We got rid of whole scales […] it definitely had an effect on our response rates and probably attrition I would think.” (TM – 3) | “For me the main contribution is that they remind us all the time what it’s about. So we don’t lose touch with what our patient population is or what the trial’s about actually.” (CI – 20) |
| “It probably made some of the questions easier to understand […] and therefore it would have improved the data collection.” (CI – 3) | “They were great […] it made it sort of like a real, researching something that was quite real and felt like it was important.” (CI - 7) |
| “The intervention acceptability, […] hopefully the effect of the intervention should hopefully be maximised by tailoring it to the needs of the community.” (CI – 1) | “I think it does make the academics stop and think […] I think that’s a good thing because I think the academics can get a bit too bogged down in their acronyms and the stats and things and actually forget that there’s people at the end of it.” (TM – 18) |
| “The ethics committee were saying that they didn’t want women to be phoned up […] the professor asked me to provide [...] a customer’s point of view […], she came back later to say that on the basis of [my letter] the ethics committee had changed their minds and now agreed that follow-up phone calls could be held […] [it] could have affected the full outcome of the trial had they not agreed to do that.” (PPI – 11-1) | “[If] academics go off into a corner developing a piece of research that's totally irrelevant to the real situation, and they never talk to patients or carers or the public about it […] the original purpose may be lost in a way; what are we doing this for?” (PPI – 22) |
