# Supplementary material for: What Difference Does Patient and Public Involvement Make and What Are Its Pathways to Impact? Qualitative Study of Patients and Researchers from a Cohort of Randomised Clinical Trials
Source: PLoS One. 2015 Jun 8;10(6):e0128817. doi: 10.1371/journal.pone.0128817 (PMC4459695; doi:10.1371/journal.pone.0128817)
Supplement: S2 File — (DOCX) [file pone.0128817.s002.docx]

**S2 APPENDIX:**

| **Table 2: - Summary of interview topics covered** | | |
| --- | --- | --- |
|  | **Researchers** | **PPI Contributors** |
| **Expectations** | Understanding of PPI | Previous experience of being a PPI contributor |
|  | Experience of including PPI in research | Expectations about what working on the current trial would be like |
|  | Goals or plans for PPI in current trial |  |
| **What happened?** | Stage of PPI implementation | How did they become involved in the trial? |
|  | Identifying and selecting PPI contributors | PPI contributor’s role |
|  | Roles of the PPI contributors | Relationship with research team |
|  | Overall experience of including PPI in the current trial |  |
| **Impact** | Perceived contributions of PPI | Differences made to the trial as a result of their input |
|  | Challenges of including PPI | Benefits to themselves of being involved |
|  |  | Challenges of being involved |
| **Training and support** | Training or support given to PPI contributors | Training or support for their role |
|  | PPI training received by researchers | Views on PPI training for researchers |
