# Supplementary material for: What Difference Does Patient and Public Involvement Make and What Are Its Pathways to Impact? Qualitative Study of Patients and Researchers from a Cohort of Randomised Clinical Trials
Source: PLoS One. 2015 Jun 8;10(6):e0128817. doi: 10.1371/journal.pone.0128817 (PMC4459695; doi:10.1371/journal.pone.0128817)
Supplement: S3 File — (DOCX) [file pone.0128817.s003.docx]

**S3** **APPENDIX:**

| **Table 3:- Informant interviewed, trial setting and intervention type** | | | | | |
| --- | --- | --- | --- | --- | --- |
| **Trial** | **CI or senior team member interviewed?** | **PPI interviewed?** | **TM interviewed?** | **Setting*** | **Intervention** |
| **1** | y | Y | N | Community | Education and exercise |
| **2** | Y | Y | N | Tertiary | Device |
| **3** | Y | Y | Y | Secondary | Education |
| **4** | Y | N | N | Tertiary | Drug |
| **5** | Y | N | Y | Secondary | Surgical |
| **6** | Y | Y | N | Secondary | Exercise |
| **7** | Y | Y | N | Primary | Community care |
| **8** | Y | Y (2 PPI contributors) | Y | Tertiary | Drug |
| **9** | Y | Y | Y | Secondary | Device |
| **10** | Y | N | N | Social care | Exercise |
| **11** | Y | Y (2 PPI contributors) | Y | Secondary | Surgical |
| **12** | Y | N | N | Secondary | Device |
| **13** | Y | N | Y | Secondary | Drug |
| **14** | Y | N | N | Secondary | Surgical |
| **15** | Y | Y | Y | Primary | Exercise |
| **16** | Y | N | N | Primary | Exercise |
| **17** | Y | N | N | Secondary | Surgical |
| **18** | Y | N | Y | Primary and secondary | Exercise and community care |
| **19** | Y | N | N | Primary | Other |
| **20** | Y | N | N | Emergency | Community care |
| **21** | Y | N | Y | Secondary | Device |
| **22** | N | Y | N | Secondary | Surgical |
| **23** | N | Y | N | Secondary | Device |
| **24** | N | Y | N | Tertiary | Drug |
| **25** | N | Y | N | Emergency | Surgical |
| **26** | N | Y | N | Secondary, Tertiary | Surgical |
| **27** | N | Y | Y | Emergency | Drug |
| **28** | N | Y | N | Secondary | Device |
| ***** A primary setting is the first point of consultation for a patient within the healthcare system, for example a general practitioner. A secondary setting is care provided by a medical specialist that cannot be directly accessed by a patient, for example as a hospital outpatient. A tertiary setting is specialist consultative healthcare, on referral from primary or secondary care, that has personnel and facilities for advanced investigation and treatment, for example a specialist cardiac unit. | | | | | |
