# Supplementary material for: What Difference Does Patient and Public Involvement Make and What Are Its Pathways to Impact? Qualitative Study of Patients and Researchers from a Cohort of Randomised Clinical Trials
Source: PLoS One. 2015 Jun 8;10(6):e0128817. doi: 10.1371/journal.pone.0128817 (PMC4459695; doi:10.1371/journal.pone.0128817)
Supplement: S4 File — (DOCX) [file pone.0128817.s004.docx]

**S4 APPENDIX:**

| **Table S4:- Examples of focussed and diffuse impact** | |
| --- | --- |
| **Focussed impact** | **Diffuse impact** |
| “We got rid of whole scales […] it definitely had an effect on our response rates and probably attrition I would think.” (TM – 3) | “For me the main contribution is that they remind us all the time what it’s about. So we don’t lose touch with what our patient population is or what the trial’s about actually.” (CI – 20) |
| “It probably made some of the questions easier to understand […] and therefore it would have improved the data collection.” (CI – 3) | “They were great […] it made it sort of like a real, researching something that was quite real and felt like it was important.” (CI - 7) |
| “The intervention acceptability, […] hopefully the effect of the intervention should hopefully be maximised by tailoring it to the needs of the community.” (CI – 1) | “I think it does make the academics stop and think […] I think that’s a good thing because I think the academics can get a bit too bogged down in their acronyms and the stats and things and actually forget that there’s people at the end of it.” (TM – 18) |
| “The ethics committee were saying that they didn’t want women to be phoned up […] the professor asked me to provide [...] a customer’s point of view […], she came back later to say that on the basis of [my letter] the ethics committee had changed their minds and now agreed that follow-up phone calls could be held […] [it] could have affected the full outcome of the trial had they not agreed to do that.” (PPI – 11-1) | “[If] academics go off into a corner developing a piece of research that's totally irrelevant to the real situation, and they never talk to patients or carers or the public about it […] the original purpose may be lost in a way; what are we doing this for?” (PPI – 22) |
